# CAP-m7G: A capsule network-based framework for specific RNA N7-methylguanosine site identification using image encoding and reconstruction layers

**DOI:** 10.1016/j.csbj.2025.02.029

**Published:** 2025-02-27

**Authors:** Peilin Xie, Jiahui Guan, Xuxin He, Zhihao Zhao, Yilin Guo, Zhenglong Sun, Lantian Yao, Tzong-Yi Lee, Ying-Chih Chiang

**Affiliations:** a Kobilka Institute of Innovative Drug Discovery, School of Medicine, The Chinese University of Hong Kong, Shenzhen, 2001 Longxiang Blvd, Longgang District, 518172, Shenzhen, China; b School of Science and Engineering, The Chinese University of Hong Kong, Shenzhen, 2001 Longxiang Blvd, Longgang District, 518172, Shenzhen, China; c School of Medicine, The Chinese University of Hong Kong, Shenzhen, 2001 Longxiang Blvd, Longgang District, 518172, Shenzhen, China; d Institute of Bioinformatics and Systems Biology, National Yang Ming Chiao Tung University, Hsinchu, Taiwan; e Center for Intelligent Drug Systems and Smart Bio-devices (IDS2B), National Yang Ming Chiao Tung University, Hsinchu, 300, Taiwan

**Keywords:** N7-methylguanosine, Bioinformatics, CGR encoding, Capsule network

## Abstract

N7-methylguanosine (m7G) modifications play a pivotal role in RNA stability, mRNA export, and protein translation. They are closely associated with ribosome function and the regulation of gene expression. Dysregulation of m7G has been implicated in various diseases, including cancers and neurodegenerative disorders, where the loss of m7G can lead to genomic instability and uncontrolled cell proliferation. Accurate identification of m7G sites is thus essential for elucidating these mechanisms. Due to the high cost of experimentally validating m7G sites, several artificial intelligence models have been developed to predict these sites. However, the performance of these models is not yet optimal, and a user-friendly web server is still needed. To address these issues, we developed CAP-m7G, an innovative model that integrates Chaos Game Representation, Capsule Networks, and reconstruction layers. CAP-m7G achieved an accuracy of 96.63%, a specificity of 95.07%, and a Matthews correlation coefficient (MCC) of 0.933 on independent test data. Our results demonstrate that the integration of Chaos Game Representation with Capsule Network can effectively capture the crucial sequence information associated with m7G sites. The web server can be accessed at https://awi.cuhk.edu.cn/~biosequence/CAP-m7G/index.php.

## Highlights

• Designed a powerful and novel interpretable framework Capsule network for identify m7G modification sites.
• RNA sequences were transformed into 2D image representations using CGR to extract potential m7G sites pattern information.
• Using Capsule Networks with Reconstruction Layers to enhance feature extraction and interpretation capabilities.
• Utilizing the comprehensive dataset enhances the model's generalizability and significantly improves performance.

## Graphical abstract

**Figure:**
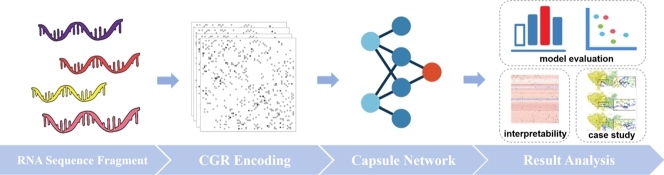

## 1 Introduction

The importance of gene expression and biological functions is fundamental to the survival and development of organisms, with post-transcriptional RNA modification acting as a key to ensure these processes function correctly [1]. N7-methylguanosine (m7G) is a highly conserved RNA modification found in various RNA molecules, including transfer RNA (tRNA) [2], microRNA (miRNA) [3], and messenger RNA (mRNA) [4]. By regulating RNA metabolism, processing, transport, and stability [5], m7G ensures proper RNA function. For instance, it stabilizes RNA secondary structures, prevents degradation, and facilitates mRNA nuclear export, thereby enhancing protein translation efficiency. [6]. Additionally, m7G also plays a key role in ribosome assembly and function, directly impacting protein synthesis [7]. These mechanisms suggest that m7G modification is significant for the physical stability of RNA and important in multiple layers of gene expression regulation. Disruption in m7G modification is linked to diseases such as cancer, neurodegenerative disorders, and metabolic conditions [8]. In cancer, the loss of m7G can lead to genomic instability, higher mutation rates, and uncontrolled cancer cell proliferation [9]. In neurodegenerative diseases, m7G dysfunction may contribute to neuronal death and pathological changes [10]. Therefore, accurate identification and analysis of m7G modification sites is essential for understanding its role in disease and developing targeted therapies.

Recent advances in high-throughput sequencing technologies have led to the development of techniques such as m7G-MeRIP-seq [11], m7G-seq [11], and AlkAniline-Seq [5] enabling precise detection of m7G modification sites across the transcriptome. These methods generate extensive experimental data, revealing m7G distribution patterns on a large scale, shedding light on its role in different biological processes. However, their high cost and time-consuming nature, limit large-scale applications.

To address these limitations, researchers have increasingly turned to computational approaches, particularly machine learning and deep learning, for predicting m7G modification sites. Chen et al. first proposed the iRNA-m7G model [12], a support vector machine (SVM)-based model [13] that integrates three sequence features, namely Nucleotide Property (NP), Pseudo Dinucleotide Composition (PseDNC) and Secondary Structure Composition (SSC). The model achieved higher accuracy in accurately identifying m7G sites than previous methods. Building on this, Liu et al. introduced m7GPredictor [14], incorporating five effective feature encoding techniques to enhance predictive performance, including PseDNC, Pseudo k-tuple composition (PseKNC), K monomeric units (K-mer), Ksnpf frequency, and NP. As algorithms evolved, Bi et al. proposed XG-m7G [15], which integrates six sequence encoding methods, binary encoding, composition of k-spaced nucleic acid pairs (CKSNAP), enhanced nucleic acid composition (ENAC), nucleotide chemical property (NCP), nucleotide density (ND), and the series correlation pseudo-dinucleotide composition (SCPseDNC), with the XGBoost algorithm to further improve accuracy. Dai et al. introduced m7G-IFL [16], an approach that integrates physical–chemical properties (PCP), ring-function-hydrogen properties (RFH), and binary and k-aggregate frequency (BKF), and iteratively refines feature representations by automatically learning the probability distribution information of sequence models. This approach significantly improved feature representation. Meanwhile, Ning et al. developed the m7G-DLSTM method [17], integrating Long Short-Term Memory (LSTM) [18] networks with fully connected layers to create a deep learning-based model for m7G prediction. More recently, TMSC-m7G model [19] introduced by Zhang et al., through the Transformer architecture, utilizes the self-attention mechanism to extract global sequence information, the model particularly excelled in long mRNA sequence m7G identification. In contrast, Zhao et al. proposed Moss-m7G [20] that combines motif detection with deep learning-based embedding modules and focuses on extracting motif features from RNA sequences. By integrating biological sequence features with deep learning embedding methods, they further improved the overall performance of m7G site identification, demonstrating a 3.09% improvement across all evaluation metrics compared to m7G-DLSTM. Each of these models offers unique strengths, contributing to the growing toolkit for predicting m7G modification sites. Table 1 summarizes all machine learning methods for predicting m7G modification sites.

**Table 1:** Summary of existing tools for m7G site identification.

Method	Feature encoding	Algorithm	Year	Reference
iRNA-m7G	NP, PseDNC, SSC	SVM	2019	[12]
m7GPredictor	PseDNC, PseKNC, K-mer, Ksnpf, NP	SVM	2020	[14]
XG-m7G	Binary, CKSNAP, ENAC, NCP, ND SCPseDNC	XGBoost	2020	[15]
m7G-IFL	PCP, RFH, BKF	XGBoost	2021	[16]
m7G-DLSTM	One-hot encoding, NP	LSTM	2021	[17]
TMSC-m7G	Multi-sense-scaled embedding	Transformer	2024	[19]
Moss-m7G	Motif	Transformer	2024	[20]

Despite significant progress in m7G site prediction, challenges remain. Most models rely on the quality and quantity of training data, yet experimental data on m7G sites are limited. This limitation affects the accuracy and robustness of the models on new datasets. In addition, current models often lack interpretability. Therefore, building more general, robust, and interpretable models is important. Although machine learning methods for m7G site prediction have improved performance, further work is needed to better identify the detailed patterns of m7G modifications and enhance accuracy.

To address these challenges, we introduce CAP-m7G, an innovative method for identifying m7G sites using image-based representations of RNA information. Our approach leverages a novel RNA encoding method known as Chaos Game Representation (CGR), a method that transforms RNA sequences into two-dimensional grayscale images, preserving sequence features in a spatial format. To analyze these representations, we further employ the Capsule Networks, an advanced next-generation artificial intelligence architecture proposed by Sabour et al. [21]. Unlike traditional convolutional neural networks, Capsule Networks capture spatial hierarchies and hidden patterns more effectively within the two-dimensional space. Previous studies have demonstrated their strong performance on benchmark datasets such as MNIST and Fashion-MNIST. Their robustness and interpretability have also gained recognition in bioinformatics, making them a promising choice for m7G site prediction [22], [23].

## 2 Materials and methods

### 2.1 Benchmark dataset

**Fig. 1:**
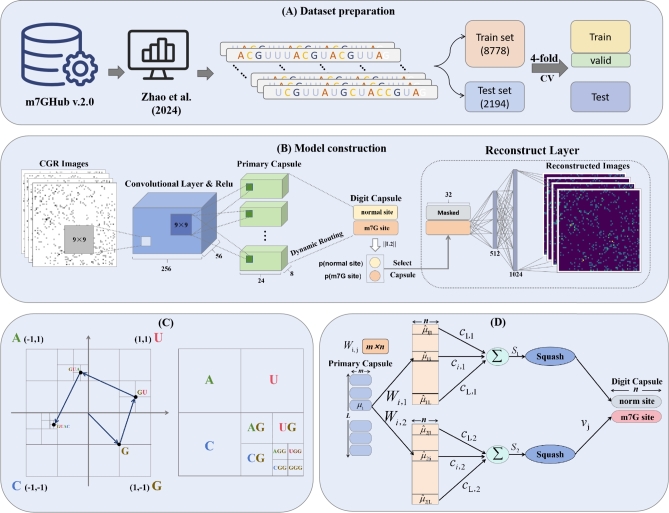
The overall workflow of the study. (A) Dataset Preparation, relevant data are collected and pre-processed for analysis. (B) m7G Model Construction Framework, illustrating the architecture of the CAP-m7G model. (C) Representation of CGR Encoding, which outlines the method used for transforming data into a format suitable for model input. (D) Dynamic Routine Process, describing the procedural steps involved in model execution and analysis.

In this study, we evaluate CAP-m7G using the experimentally valida dataset collated by Zhang et al. [20] from m7GHub v.2.0 [24]. The dataset is processed using the CD-HIT program [25] with a similarity threshold of 0.8, resulting in 5,486 high-quality sequences centered around m7G modification sites, each extending 501 bp. This represents a significant enhancement over previous datasets, which contained only 741 m7G modification records [14], providing a more comprehensive and reliable resource for model training and validation. To ensure a balanced dataset, we randomly select an equal number of non-m7G sites and their flanking regions from the human genome as negative samples. The dataset is then split into training and testing sets at a ratio of 8:2. The training set undergoes four-fold cross-validation, which segments the data further segmenting into training and validation subsets to fine-tune the model performance. This rigorous validation framework enhances the model's reliability and prepares CAP-m7G for real-world applications in m7G modification site prediction. Fig. 1A illustrates the data preparation process.

### 2.2 The overall framework of CAP-m7G

The architecture of the CAP-m7G model is depicted in Fig. 1B. The process begins with CGR encoding of RNA sequences, converting them into a 64×64 grayscale image. This image undergoes an initial feature extraction through a two-dimensional convolutional neural network (Conv2D), to capture essential sequence patterns. The extracted features are then fed into a capsule network, which further extracts higher-order features and models the spatial relationships among RNA sequences within the CGR image representation. Finally, the output vectors from the capsule network pass through a reconstruction layer, which regenerates the CGR image, thereby enhancing the coherence of the vector space representation.

### 2.3 Chaos game representation

CGR is a technique for mapping biological sequences into two-dimensional images. It is widely applied in the study of DNA, RNA, and proteins [26]. By transforming sequences into a set of points in two-dimensional space, the distribution and positions of these points reflect the characteristics and patterns of the sequences. This method provides an intuitive representation to reveal complex patterns and structures within sequences. For instance, motifs appear as dense clusters of points or recurring patterns in the two-dimensional image, providing a visual representation that facilitates in-depth bioinformatic analysis and the identification of functional features. Consequently, CGR encoding has become an essential tool for sequence representation and computational biology.

In this study, CGR encoding was utilized to convert RNA sequences into a two-dimensional graphical representation. The four nucleotides-adenine (A), uracil (U), cytosine (C), and guanine (G)-were each assigned to a vertex of a square. For an RNA sequence *S* of length n ($S=s_{1},s_{2},...,s_{n}$), where each nucleotide $s_{i}$ belongs to the set A,U,C,G, it is mapped to a specific point in a two-dimensional coordinate system. The corresponding position $\left(x_{i},y_{i}\right)$ for each nucleotide $s_{i}$ is determined based on its base type and its association with the preceding nucleotide. The coordinates $\left(x_{i},y_{i}\right)$ for each nucleotide are defined as follows:

(1) $$\left(x_{i},y_{i})=\frac{1}{2}((x_{i-1},y_{i-1})+h(s_{i})\right)$$

where $\left(x_{0},y_{0})=(0,0\right)$ and

(2) $$h(s_{i})=\left\{ \begin{array}{c} (-1,1),s_{i}=A\\ (1,1),s_{i}=U\\ (-1,-1),s_{i}=C\\ (1,-1),s_{i}=G \end{array}\right.$$

Fig. 1C illustrates an RNA sequence encoded using CGR, showcasing the segmentation of the CGR space as it advances through the iterative encoding procedure.

The generalized form of CGR, known as Frequency Matrix Representation (FCGR), transforms sequences of varying lengths into uniformly sized images or matrices [26]. Unlike traditional CGR, FCGR divides the CGR space into an N × N grid and counts k-mer frequencies within each region. This approach produces a matrix that encodes k-mer distributions without relying on precise positional information, offering a more compact and resilient representation of sequence data. By capturing motif-like patterns, FCGR enables the detection of underlying sequence structures, making it a powerful tool for genomic or protein sequence analysis.

In this study, we utilize N=64 to generate images with dimensions $R^{64\times 64}$ based on the FCGR method.

### 2.4 Capsule network with reconstruction layer

Capsule networks, a novel deep learning framework proposed by Sabour et al. [21], aim to overcome key limitations of Convolutional neural networks (CNNs). While CNNs have achieved remarkable success across various domains, they still have notable drawbacks. In particular, their reliance on pooling operations often results in the loss of crucial spatial information, limiting their ability to capture spatial hierarchies between features. Capsule networks address these challenges by introducing capsules as fundamental building blocks. Each capsule represents a vector, encoding information about specific entities, in contrast to the scalar outputs produced by traditional neural networks. The capsule's direction reflects the entity's characteristics, while its magnitude corresponds to the likelihood of the entity's presence. The conventional capsule network architecture comprises two layers: the Primary capsule layer and the Digit capsule layer. Expanding on this design, we incorporate a reconstruction layer to further enhance feature extraction ability and improve overall model performance.

As shown in Fig. 1B, the Primary capsule layer employs a Conv2D layer to enhance feature extraction. The resulting outputs are then transformed into multiple *m*-dimensional vectors, where *m* is a tunable hyperparameter. These vectors then undergo a non-linear ‘squash’ function (Equation (3)), which normalizes their magnitudes to a range of [0, 1] while preserving their directional information.

(3) $$squash(s)=\frac{{\left\|s\right\|}^{2}}{1+{\left\|s\right\|}^{2}}\frac{s}{\left\|s\right\|}$$

Fig. 1D further illustrates the computational steps between the Primary and Digit capsule layers. The output vectors $u_{i}$ from the Primary capsule layer are first multiplied by a learnable weight matrix $W_{i,j}$ generating the prediction vectors ${\overset{ˆ}{u}}_{j|i}$. The next step involves computing $S_{j}$, the weighted sum of all the resulting ${\overset{ˆ}{u}}_{j|i}$ vectors, as defined by the following equation:

(4) $${\overset{ˆ}{u}}_{j|i}=W_{i,j}u_{i}$$

(5) $$S_{j}=\sum_{i=1}^{L}c_{i,j}{\overset{ˆ}{u}}_{j|i}$$

Where, *i* and *j* denote capsules in the Primary and Digit capsule layers, respectively, while *L* represents the total number of Primary capsules. The coupling coefficients $c_{i,j}$, computed using the dynamic routing algorithm (Supplementary Algorithm 1), reflect the coupling strength between the primary capsule *i* and the digit capsule *j*. The vector $S_{j}$ is then processed through the Squash function, producing an output vector $v_{j}$ with a length constrained to the range [0, 1].

In capsule network theory, the vector $v_{j}$ serves to distinguish between positive and negative samples. In this context, it specifically differentiates m7G sites from normal sites. Each element of $v_{j}$ captures features relevant to either positive or negative samples. The Euclidean length of $v_{j}$ (L2 norm) yields the probability that a given sample corresponds to an m7G site or a normal site. That is,

(6) $$p_{j}={\left\|v_{j}\right\|}_{2}$$

where $p_{j}$ respectively denote the model's predicted probability of a site being either an m7G site or a normal site.

The output vector $v_{j}$ from the Digit capsule layer is selected based on its probability $p_{j}$. The capsule with the highest $p_{j}$ is chosen and passed to the reconstruction layer, which leverages the selected capsule's features to regenerate a corresponding CGR image. This process not only enhances the capsule network's feature extraction capabilities, but also improves model interpretability by visualizing the extracted features. As a result, the network learns more representative features, reducing the likelihood of misclassification.

The model is optimized by integrating margin loss and reconstruction loss. Margin loss enhances the model's discriminative ability by maximizing the activation of capsules corresponding to the correct class while suppressing those associated with incorrect classes. The margin loss for the capsule network is formulated as follows:

(7) $$L_{margin}=T_{k}\cdot \max(0,m^{+}-\|v_{k}{\left\|\right)}^{2}+\lambda (1-T_{k})\cdot \max(0,\|v_{k}{\left\|-m^{-}\right)}^{2}$$

where $T_{k}$ is a binary indicator (0 or 1) that denotes whether class *k* is the correct class. $\left\|v_{k}\right\|$ represents the L2 norm of the output vector for class *k*. $m^{+}$ and $m^{-}$ denote the upper and lower margins, respectively, while *λ* is a down-weighting parameter when $T_{k}$ = 0 to balance the loss. The second component, reconstruction loss, ensures that the activated capsules can accurately regenerate the original CGR image. This process enhances the significance of the output vectors, allowing the network to capture essential sample features and further improving its generalization ability. The reconstruction loss is defined as follows:

(8) $$L_{reconstruction}=\frac{1}{N}\sum_{i=1}^{N}{\left(CGR_{i}-{\overset{ˆ}{CGR}}_{i}\right)}^{2}$$

where *N* represents the total number of pixels in the image, $CGR_{i}$ denotes the value of the *i*-th pixel in the original CGR image, and ${\overset{ˆ}{CGR}}_{i}$ is the value of the *i*-th pixel in the reconstructed image.

### 2.5 Performance evaluation and pipeline construction

This study evaluates the model's performance using Accuracy, Sensitivity, Specificity, and the Matthews Correlation Coefficient (MCC). These metrics are defined as follows:

(9) $$Accuracy=\frac{TP+TN}{TP+TN+FP+FN}$$

(10) $$Recall=\frac{TP}{TP+FN}$$

(11) $$Specificity=\frac{TN}{TN+FP}$$

(12) $$F1-score=2\times \frac{TP}{2TP+FN+FP}$$

(13) $$MCC=\frac{TP\times TN-FP\times FN}{\sqrt{\left(TP+FP)(TP+FN)(TN+FP)(TN+FN\right)}}$$

where TP, TN, FP, and FN denote the number of true positives, true negatives, false positives, and false negatives, respectively. AUC is defined as the area under the Receiver Operating Characteristic (ROC) curve, with values ranging from 0.5 to 1. In this study, both ROC and Precision-Recall (PR) curves, along with their respective AUC values, were used to provide a comprehensive assessment of model performance. The CAP-m7G method utilized the “Kaos” R package to generate CGR encodings for RNA sequences.

In the present study, four-fold cross-validation was employed on the training set to optimize model parameters and assess preliminary performance. The training data was divided into four equal folds, ensuring that the training and validation splits were consistent across all comparative methods. For each fold, input sequences of 501 bp were utilized, and experimental parameters were configured in strict accordance with the guidelines provided for each respective tool. Following cross-validation, the optimal parameter configuration was selected, and the final model was comprehensively evaluated on an independent test set to determine its overall performance. The cross-validation results are given in Table S3.

The model is trained for 100 epochs using the Adam optimizer [27] with an initial learning rate of 0.001. The implementation is built in PyTorch [28], and training is conducted on four Nvidia 2080 Ti GPUs.

## 3 Results and discussion

### 3.1 Performance comparison with existing methods

In the standard binary classification task for m7G site prediction, the CAP-m7G model is rigorously compared against multiple existing methods, as presented in Table 2. The results demonstrate that CAP-m7G outperforms competing approaches, achieving significant improvements in accuracy, recall, specificity, MCC, and AUC. Notably, CAP-m7G attains an accuracy of 96.63%, reflecting an 12.27% increase over its closest competitor.

**Table 2:** Benchmark performance comparison between CAP-m7G and other existing methods. The best performances are marked in bold.

Method	ACC	Recall	Specificity	F1	MCC	AUC
iRNA-m7G(101)	0.7881	0.7821	0.7940	0.7868	0.5762	0.8842
iRNA-m7G(501)	0.6609	0.8687	0.4531	0.7192	0.3538	0.8198
m7GPredictor(101)	0.8140	0.7912	0.8368	0.8097	0.6287	0.9018
m7GPredictor(501)	0.7475	0.7010	0.7940	0.7352	0.4971	0.8298
m7G-DLSTM	0.8154	0.8177	0.8131	0.8158	0.6308	0.8907
Moss-m7G	0.8436	0.8231	0.8641	0.8403	0.6879	0.9235
CAP-m7G	**0.9663**	**0.9817**	**0.9507**	**0.9668**	**0.9330**	**0.9950**

Note: The numbers 101 and 501 indicate the default input length, and other models use a default input length of 501 bp.

In terms of MCC, CAP-m7G outperforms the second-ranked method by 0.16, achieving a value of 0.9330. This indicates that CAP-m7G excels at identifying positive samples, and accurately excludes negative samples. Furthermore, CAP-m7G achieves outstanding results in both recall (98.17%) and specificity (95.07%).

The strengths of CAP-m7G arise from its unique model architecture, which integrates the features of CGR encoding with capsule networks to effectively capture and model complex RNA sequence features. CGR encoding transforms RNA sequences into images, preserving global information in a structured format. The capsule network then models the spatial relationships between nucleotides within the CGR image, extracting higher-order features that enhance sequence representation. This design significantly improves the depth and accuracy of feature extraction, enabling the model to better handle complex sequence relationships. Finally, the output vectors pass through a reconstruction layer, generating refined CGR images that enhance the coherence of vector space representation and preserve the semantic integrity of the extracted features.

In summary, CAP-m7G exhibits outstanding performance across various metrics, underscoring its superiority in m7G site prediction. Its exceptional accuracy stems from its innovative model architecture and its ability to effectively model feature space relationships, making it a powerful tool for RNA modification analysis.

### 3.2 The effectiveness of the capsule network architecture

**Fig. 2:**
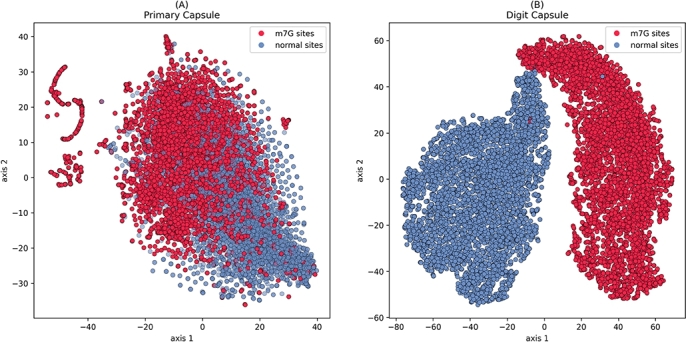
Visualization of m7G sites alongside training sites within the Primary Capsule and Digit Capsule layers of CAP-m7G. (A) Primary capsule layer. (B) Digit capsule layer.

To further validate the effectiveness of the capsule network architecture, we first extract sample features from the Primary Capsule and Digit Capsule layers using the training set. We then apply t-Distributed Stochastic Neighbor Embedding (t-SNE) for dimensionality reduction, compressing the high-dimensional feature space into two dimensions to better visualize the sample distribution. This approach enables a clear distinction between m7G sites and normal sites. The resulting scatter plot, shown in Fig. 2, represents m7G sites as red points and normal sites as blue points represent normal sites.

As shown in Fig. 2A, positive and negative samples appear mixed with no clear separation before processing with the capsule network. In contrast, Fig. 2B reveals a well-separated distribution after processing, forming a more distinct boundary that enhance sample recognizability between samples. This demonstrates that the capsule network architecture effectively processes features, making differences between positive and negative samples more pronounced and thereby improving the model's classification performance. The model's exceptional performance primarily stems from the dynamic routing algorithm within the capsule network, which enables flexible information flow between capsules and dynamically adjust connection weights. This dynamic routing mechanism significantly enhances the network's ability to capture spatial relationships between features, highlighting the practical potential of capsule networks in bioinformatics applications.

### 3.3 Ablation study

**Fig. 3:**
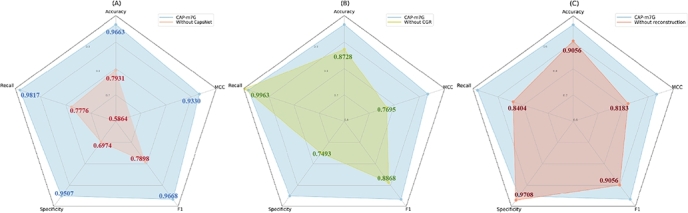
Results of the ablation experiment: (A) without CGR encoding, (B) without the CapsNet architecture, and (C) without the reconstruction layer. Each axis represents a specific performance metric, illustrating the impact of removing each component on the model's overall effectiveness.

To assess the contributions of different components in CAP-m7G, we conduct ablation analysis. The results, as depicted in Fig. 3, show that CAP-m7G achieved outstanding performance across multiple metrics, including accuracy (96.63%), recall (98.17%), specificity (95.07%), F1 score (96.68%), and MCC (0.9330). Replacing the capsule network with CNN and MLP layers of comparable parameters, denoted as without CapsNet, performance deteriorates significantly. The accuracy drops to 79.31%, and both recall and specificity decline significantly. This emphasizes the critical role of the capsule network in capturing spatial relationships between features.

When CGR encoding is replaced with one-hot encoding of the AUCG, denoted as without CGR encoding, the model's accuracy drops to 87.28%, with a notable decrease in specificity to 74.93%. This result underscores the importance of CGR in effective feature representation. Additionally, omitting the reconstruction process results in an accuracy of 90.56%, along with a decline in the F1 score and a MCC of 0.8183. These findings demonstrate that the reconstruction mechanism refines feature representations and enhancing overall performance. The ablation study confirms that the capsule network architecture, CGR encoding, and reconstruction process are essential for optimizing m7G site recognition.

**Fig. 4:**
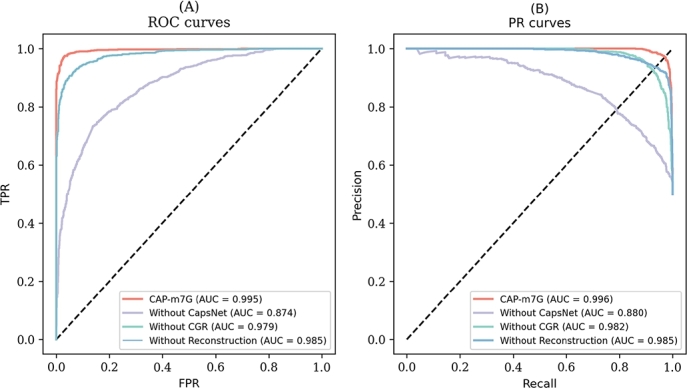
(A) ROC and (B) PR curves from ablation experiments evaluating CAP-m7G. The comparisons include the base model and its variant versions, each omitting a key component: without CGR encoding (Without CGR), without the Capsule Network architecture (Without CapsNet), and excluding the reconstruction layer (Without Reconstruction). These comparisons elucidate the contributions of each component to the model's overall performance.

To further evaluate the performance of CAP-m7G, we plot ROC and PR curves for both the CAP-m7G model and its variants without key components, as illustrated in Fig. 4. The ROC curves indicate that CAP-m7G consistently outperforms models without CAPsNet, without CGR encoding, or without reconstruction layers. This is evident from its larger area under the curve (AUC), reflecting an enhanced ability to distinguish between positive and negative samples. Similarly, the PR curves further demonstrate CAP-m7G's advantage, with a higher area under the PR curve compared to alternative models. These results confirm that the capsule network architecture, CGR encoding, and reconstruction process are crucial in improving predictive abilities for m7G recognition. Overall, our results demonstrate the robustness of CAP-m7G in identifying m7G sites.

### 3.4 Feature cluster analysis

The construction of a robust classifier depends on the effective extraction of discriminative features. CAP-m7G achieves precise differentiation of RNA modification sites through its unique feature extraction method. Specifically, it utilizes CGR encoding to convert RNA sequences into 64×64 grayscale images, enabling efficient feature extraction via a capsule network architecture. Within the capsule network, the Digit Capsule layer consists of two capsules, each represented as a 32-dimensional vector, corresponding to normal sites and m7G sites, respectively. This design enhances the model's ability to capture and differentiate distinctive features between m7G sites and normal sites, improving classification performance.

**Fig. 5:**
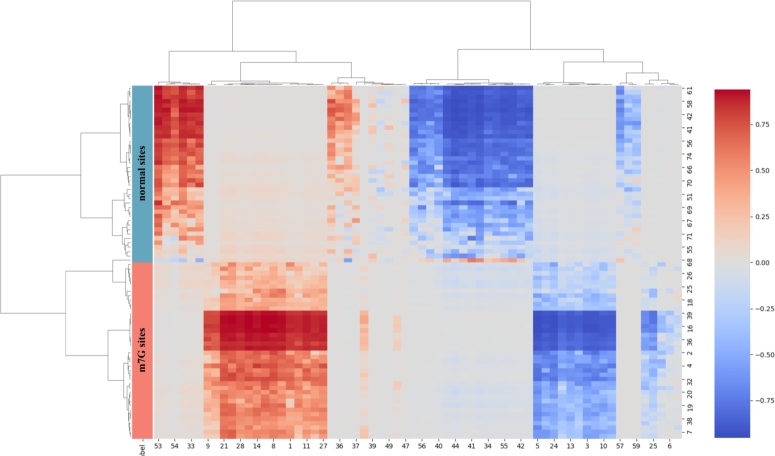
The clustering analysis map of latent features generated by CAP-m7G on the independent test set.

To further evaluate CAP-m7G's ability to extract discriminative features for m7G sites, we randomly select 40 m7G sites and 40 normal sites from the test set for feature clustering analysis. We employ hierarchical clustering with complete linkage, as it effectively preserves similarity and hierarchical structure among samples. The clustering results, shown in Fig. 5, reveal that m7G sites and normal sites are clearly divided into distinct branches, demonstrate that the features extracted by CAP-m7G can effectively distinguish m7G sites.

### 3.5 Significance analysis

**Fig. 6:**
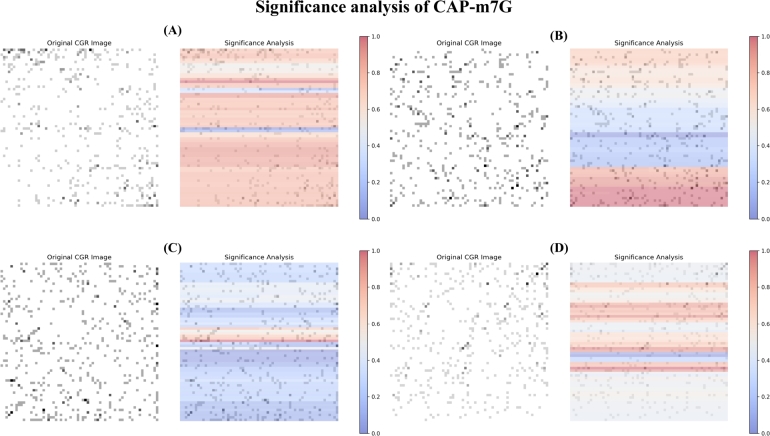
Significance analysis of CAP-m7G. (A-B) represent m7G sites, while (C-D) correspond to normal sites. For each pair, the left image displays the original CGR image, while the right image is the corresponding significance image. In the significance images, regions shaded closer to red indicate features that positively influence the model's predictions, whereas areas shaded closer to blue represent features that negatively impact the model's predictions.

To analyze our model, we randomly select four samples and use the SHAP explainer to perform significance analysis with a 9x9 mask matrix. As shown in Fig. 6, subfigures A and B correspond to m7G site samples, while subfigures C and D represent normal site samples. In each subfigure, the left panel displays the original CGR image, while the right panel presents the significance analysis heatmap, where red-highlighted regions indicate areas with a greater positive influence on the model's predictions. It is evident that there are distinct differences between m7G and normal site samples. For m7G site samples, the upper and lower regions of the image, particularly the CG-rich areas at the bottom, play a crucial role in the model's decision-making. In contrast, for normal site predictions, the central region of the image exhibits higher significance. Notably, the significance analysis of CAP-m7G aligns closely with previous motif analysis of m7G sites [20], where motifs such as base “G”, particularly the patterns “GGG”, “CU”, and “UC”, frequently appear. Furthermore, motif analysis using MEME [29] on our m7G dataset produced similar results (see Fig. S1), thereby corroborating these findings. This consistency further supports CAP-m7G's ability to capture hidden sequence patterns upstream and downstream of m7G sites. We extend this analysis to other m7G and normal site samples. The results are presented in Fig. S2.

### 3.6 Case study

**Fig. 7:**
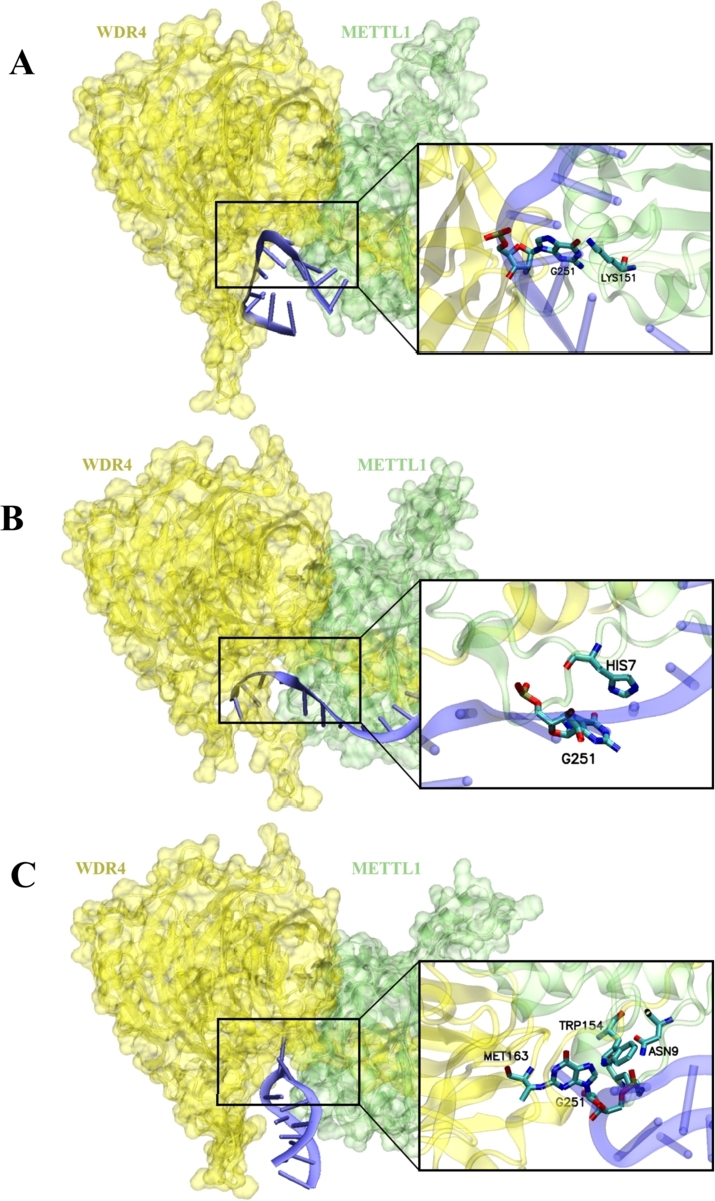
Structures of Protein-RNA complexes predicted by AlphaFold3. The WDR4 protein is highlighted in yellow, while METTL1 is shown in green. Truncated RNA sequences are depicted in blue, with the guanine nucleotide (G) emphasized. Its immediate environment within a 3 Å radius is visualized using a bond representation model, highlighting the molecular interactions occurring at this site.

Previous studies have shown that methyltransferase-like 1 (METTL1) and WD repeat domain 4 (WDR4) protein complex can bind to RNAs and catalyze their methylation [11], [30]. This suggests that the MTTTL1-WDR4 protein complex can recognize the m7G site. To model this, we employed AlphaFold3 [31] to construct the structures that contain the METTL1-WDR4 complex with three different m7G RNA sequences, each consisting of 11 bases with guanosine at the center. The predicted complex structures are highly confident. The predicted local distance difference test (pLDDT) score exceeds 90 for most of the proteins and ranges between 50 and 90 around the binding site, while the interface predicted Template Modeling (ipTM) score is all above 0.85. The predicted structures demonstrate that the METTL1-WDR4 complex can indeed recognize the guanosine at an m7G site, through significant hydrogen-bond interactions between the guanosine and the protein complex (Fig. 7) [32]. This provides crucial molecular insights to support our computational predictions.

### 3.7 CAP-m7G web interface

**Fig. 8:**
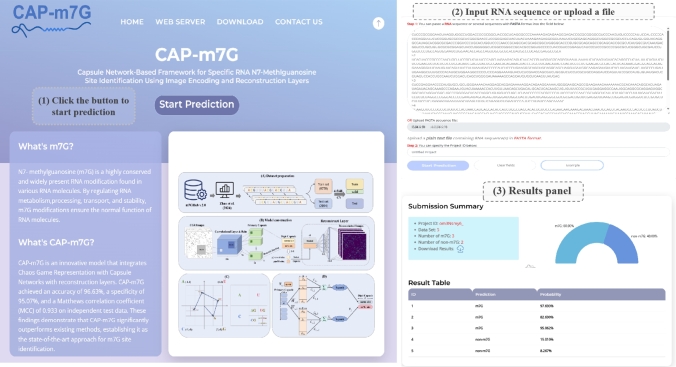
Demonstration of the CAP-m7G web interface with the example sequences.

A user-friendly web interface was developed to facilitate access to CAP-m7G, available at https://awi.cuhk.edu.cn/~biosequence/CAP-m7G/index.php. As shown in Fig. 8, users can initiate predictions by selecting “Start Prediction” on the homepage. This navigates to the prediction page, where RNA sequences in FASTA format can be entered directly or uploaded. The prediction process is activated by clicking the dedicated prediction button. Results are then displayed on a separate page. The output is divided into two sections: a “Prediction Summary” that provides an overview of the numbers of m7G and normal sites with a data download option, and a “Prediction Results” section that displays the detailed outcomes for each sequence, including prediction probabilities. This design ensures that researchers can efficiently perform high-throughput m7G site predictions with ease.

## 4 Conclusion

In this study, we introduce CAP-m7G, a model specifically designed for RNA m7G site prediction. By leveraging image-based representations of RNA sequences as input, CAP-m7G provides an intuitive visualization of sequence patterns. The integration of CGR, capsule network, and reconstruction layers successfully captures the hidden sequence patterns around m7G sites. As a result, CAP-m7G achieves state-of-the-art performance, demonstrating significant improvements across multiple metrics in m7G site identification. This research applies computer vision techniques to process sequence data, offering novel insights into the prediction of RNA modification sites.

## Funding

This work was supported by 10.13039/501100010877Shenzhen Science and Technology Innovation Commission (JCYJ20230807114206014), and the Kobilka Institute of Innovative Drug Discovery, 10.13039/100022813The Chinese University of Hong Kong, Shenzhen, China. This work was also financially supported by the Center for Intelligent Drug Systems and Smart Biodevices (IDS2B) from The Featured Areas Research Center Program within the framework of the Higher Education Sprout Project and Yushan Young Fellow Program (113C51N055) by the 10.13039/100010002Ministry of Education (MOE) and 10.13039/100020595National Science and Technology Council (NSTC 113-2321-B-A49-025-, 113-2634-F-039-001, 113-2221-E-A49-160-MY3 and 112-2740-B-400-005) in Taiwan and The 10.13039/501100004737National Health Research Institutes (NHRI-EX114-11320BI) in Taiwan.

## CRediT authorship contribution statement

**Peilin Xie:** Writing – original draft, Visualization, Methodology, Formal analysis, Conceptualization. **Jiahui Guan:** Writing – review & editing, Formal analysis, Data curation, Conceptualization. **Xuxin He:** Writing – review & editing, Visualization, Validation. **Zhihao Zhao:** Writing – review & editing, Visualization, Validation. **Yilin Guo:** Writing – review & editing, Validation. **Zhenglong Sun:** Supervision. **Lantian Yao:** Writing – review & editing, Project administration, Conceptualization. **Tzong-Yi Lee:** Writing – review & editing, Supervision, Funding acquisition. **Ying-Chih Chiang:** Writing – review & editing, Supervision, Funding acquisition, Conceptualization.

## Declaration of Competing Interest

The authors declare that they have no known competing financial interests or personal relationships that could have appeared to influence the work reported in this paper.

## Data Availability

The data used for training and testing the models in this study and the source code files for reproducing and evaluating CAP-m7g are available at https://github.com/Cpillar/CAP-m7G.

## Appendix A Supplementary material

MMC This document provides detailed descriptions of the experimental parameters and cross-validation outcomes, a comprehensive step‐by‐step account of the dynamic routing algorithm, and thorough results from both motif analysis and case study scoring. MMC
