# Supplementary material for: CAP-m7G: A capsule network-based framework for specific RNA N7-methylguanosine site identification using image encoding and reconstruction layers
Source: Comput Struct Biotechnol J. 2025 Feb 27;27:804–12. doi: 10.1016/j.csbj.2025.02.029 (PMC11919597; doi:10.1016/j.csbj.2025.02.029)

**SUPPLEMENTARY MATERIALS**

**Table S1. The table of Ablation result**

**Table S2. The Hyperparameters for CAP-m7G**

**Table S3. Four-fold cross-validation prediction results**

**Figure S1. Motif analysis of m7G sequence**

**Figure S2. Significant result of m7G site and normal site**

**Figure S3. Alphafold3 result**

**Algrithm S1. Routing algorithm**

**Table S1. The table of Ablation result**

| Method | Accuracy | Recall | Specificity | AUC | F1 | MCC |
| --- | --- | --- | --- | --- | --- | --- |
| CAP-m7G | **0.9663** | 0.9817 | 0.9507 | **0.995** | **0.9668** | **0.9329** |
| Without CapsNet | 0.7931 | 0.7776 | 0.6974 | 0.874 | 0.7898 | 0.5864 |
| Without CGR | 0.8728 | **0.9963** | 0.7493 | 0.979 | 0.8868 | 0.7695 |
| Without reconstruction | 0.9056 | 0.8404 | **0.9708** | 0.985 | 0.9056 | 0.8183 |

**Table S2. The Hyperparameters for CAP-m7G**

| **Hyperparameters** | **Search space** | **Best hyperparameters** |
| --- | --- | --- |
| **Size of CGR image** | **{16, 32, 64, 128}** | **64** |
| **Kernel size of Conv** | **{3,5,7,9}** | **9** |
| **Number of primary capsule** | **{4,8,16,32}** | **16** |
| **Number of routing** | **{2,3,4}** | **3** |
| **Dimension of type capsule** | **{8,16,32,64}** | **32** |
| **Number of epochs** | **{50,100,150,200}** | **100** |
| **Learning rate** | **{0.1,0,01,0.001,0.0001}** | **0.001** |

**Table S3. Four-fold cross-validation prediction results**

| Method | ACC | Recall | SPEC | F1 | MCC | AUC |
| --- | --- | --- | --- | --- | --- | --- |
| m7GPredictor | 0.7603±0.0092 | 0.7612 | 0.7593 | 0.7604 | 0.5205 | 0.8387 |
| m7G-DLSTM | 0.8159±0.0037 | 0.8277 | 0.8040 | 0.8180 | 0.6319 | 0.9023 |
| Moss-m7G | 0.8409±0.0028 | 0.8013 | 0.8806 | 0.8339 | 0.6840 | 0.9188 |
| **CAP-m7G** | **0.9622±0.0046** | **0.9581** | **0.9584** | **0.9620** | **0.9244** | **0.9873** |

**Figure S1. Motif analysis of m7G sequence**

**
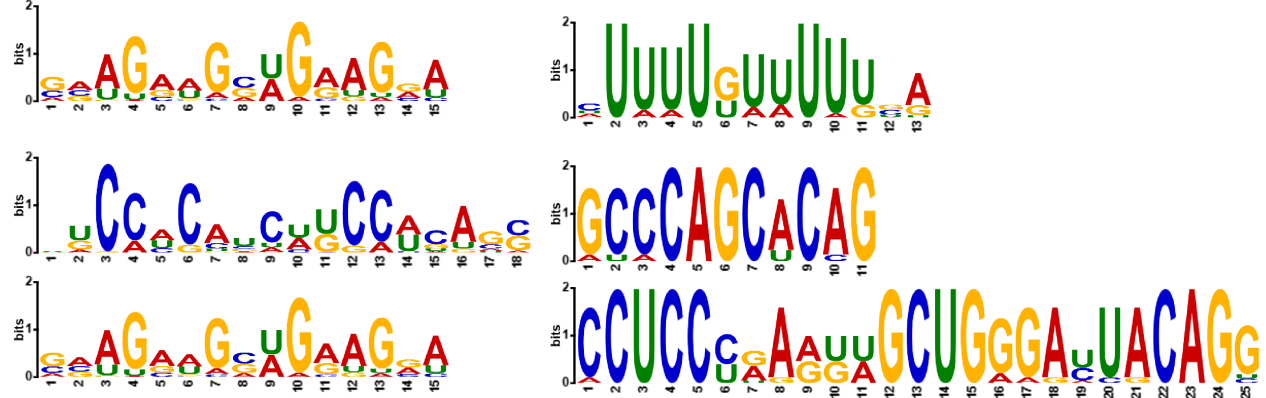
Figure S2. Significant result of m7G site and normal site**

**
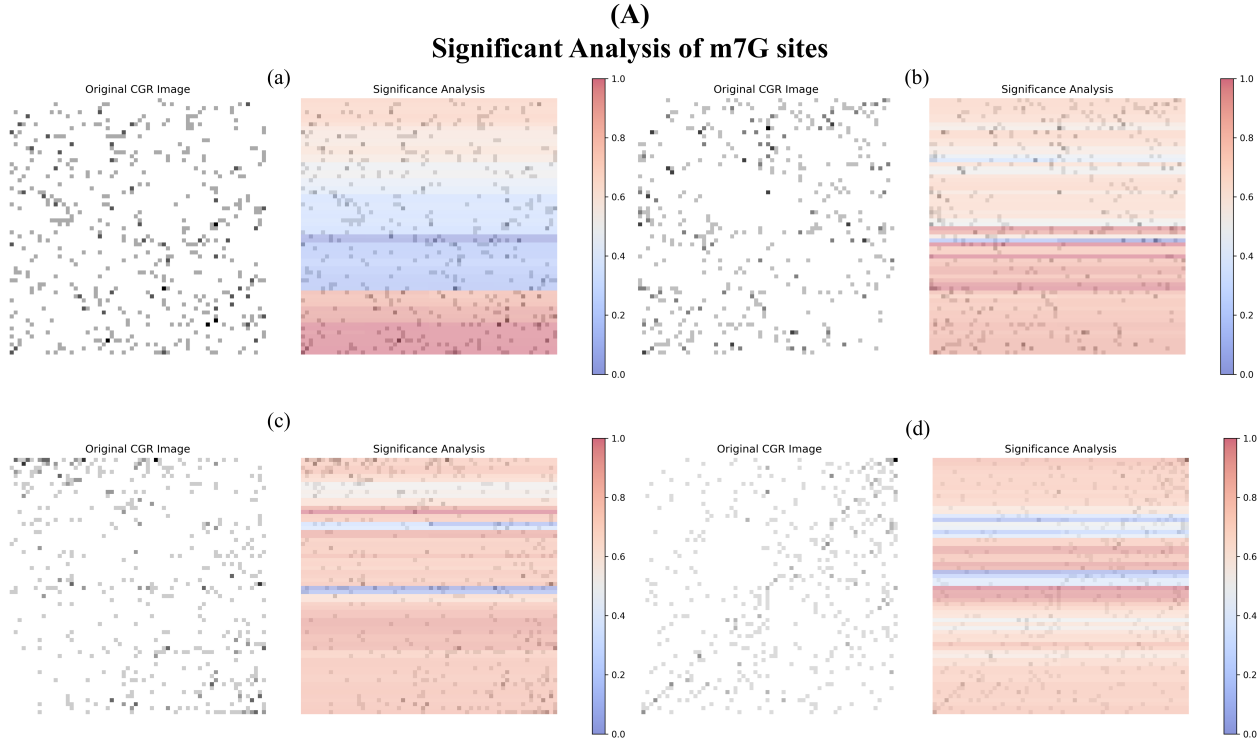
**

**
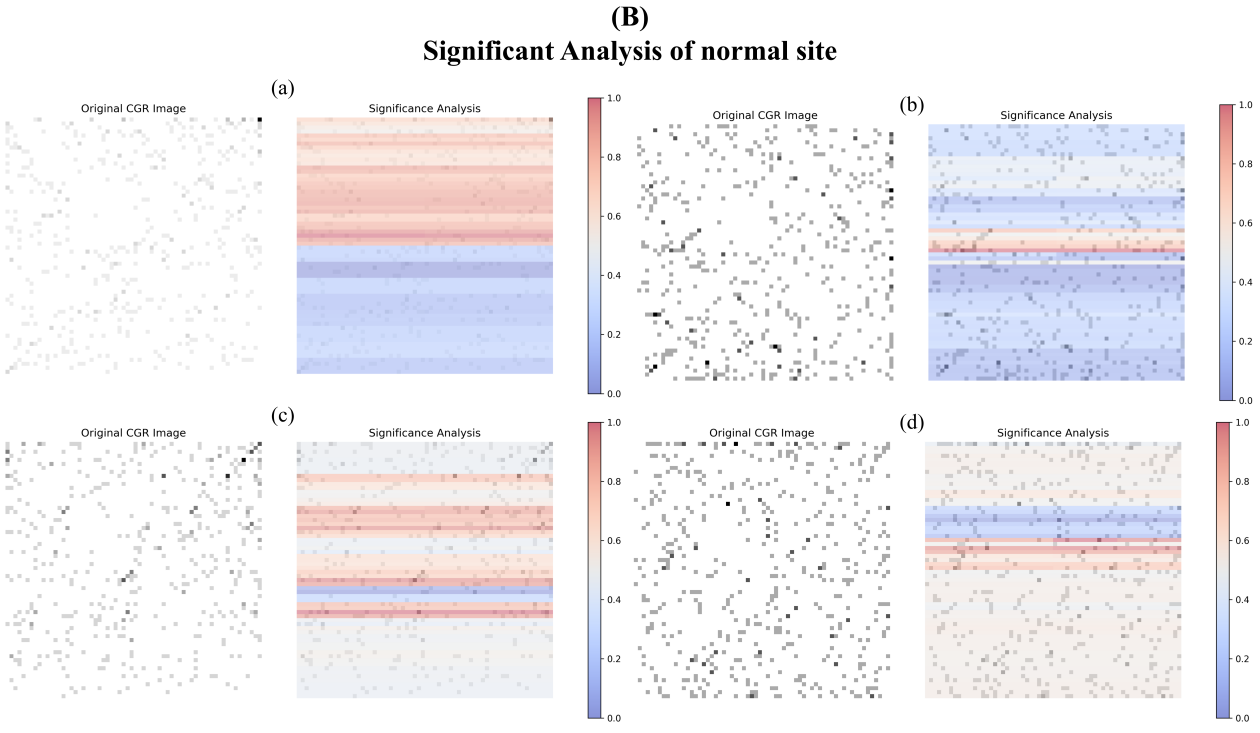
**

**Figure S3. Alphafold3 result**

pLDDT score distribution in the AlphaFold3-predicted METTL1-WDR4-RNA complex in Figure 7. The structure is colored according to the pLDDT (predicted Local Distance Difference Test) scores.


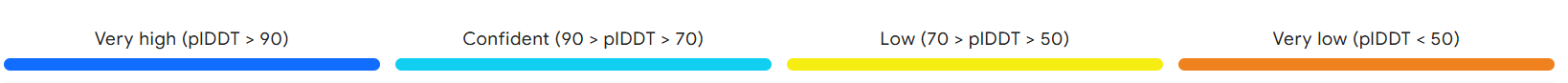

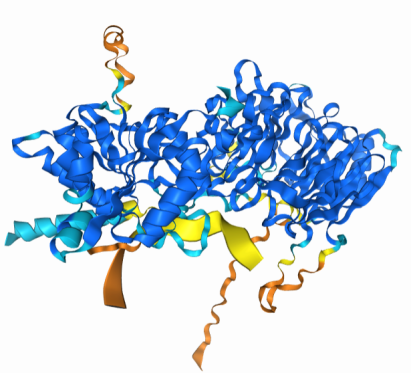

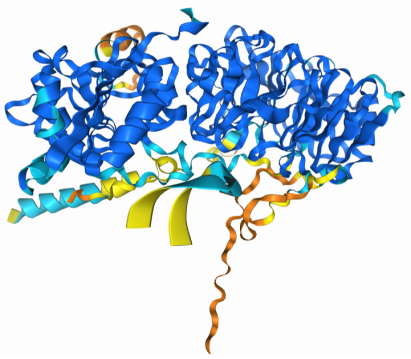

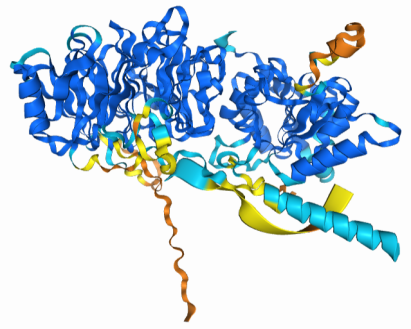


**Algrithm S1. Routing algorithm**


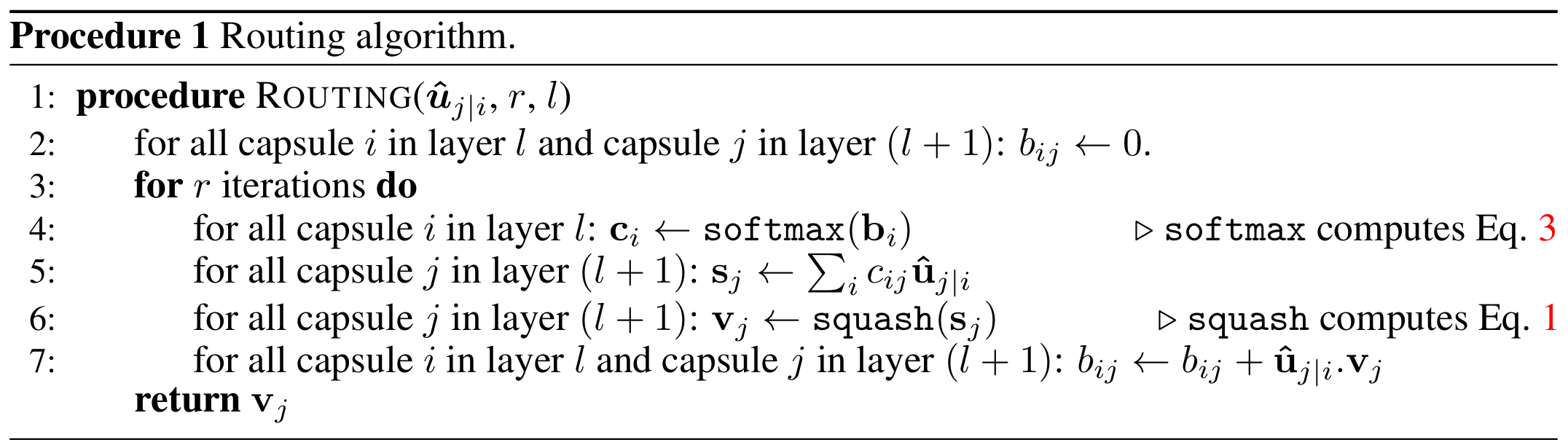

Supplement: MMC — This document provides detailed descriptions of the experimental parameters and cross-validation outcomes, a comprehensive step‐by‐step account of the dynamic routing algorithm, and thorough results from both motif analysis and case study scoring. [file mmc1.docx]
